# Severe Acute Respiratory Distress Syndrome after Laparoscopic Appendectomy in a Young Adult

**DOI:** 10.7759/cureus.1664

**Published:** 2017-09-08

**Authors:** Gabriel O Ologun, Daniel Ridley, Nhean D Chea, Daniel Golden, Anne Lihau-N'Kanza, Philip McPhail

**Affiliations:** 1 General Surgery, Guthrie Clinic/Robert Packer Hospital; 2 Medical Student, Geisinger Commonwealth School of Medicine; 3 Trauma/critical Care, Guthrie Clinic/Robert Packer Hospital

**Keywords:** appendicitis, mechanical ventilation, acute respiratory distress syndrome (ards), intensive care unit(icu), systemic inflammatory response syndrome (sirs), sepsis

## Abstract

Severe acute respiratory distress syndrome after laparoscopic appendectomy for acute appendicitis in a young adult is an uncommon complication. We describe the case of a 25-year-old male who developed severe acute respiratory distress syndrome after an uneventful laparoscopic appendectomy for a perforated appendix, requiring mechanical ventilation in the intensive care unit.

## Introduction

Acute respiratory distress syndrome (ARDS) is a diffuse, sudden-onset inflammatory process involving both lungs that results in increased lung pulmonary vascular permeability, decreased lung compliance, and alveolar edema and damage [1]. Symptoms usually present within six to 72 hours after an inciting event, and generally tend to worsen rapidly [2]. ARDS is more common in older people, with an incidence of 306 per 100,000 person-years among individuals ages 75 to 84 compared to 16 per 100,000 person-years among individuals ages 15 to 19 [3]. Here, we present an uncommon case of severe acute respiratory distress syndrome (ARDS) after a laparoscopic appendectomy for acute appendicitis in a young adult. Informed consent was obtained for the case report, images and for publication.

## Case presentation

The patient is a 25-year-old male with a history of mild asthma who presented to the emergency department (ED), with a one-day history of acute onset abdominal pain on the right side of his abdomen. The severity of abdominal pain had increased over time and was associated with nausea, vomiting, and fever. His past surgical history was notable for tonsillectomy for chronic tonsillitis. He denied a history of smoking. He last used his albuterol inhaler over eight years ago.

**Figure 1:**
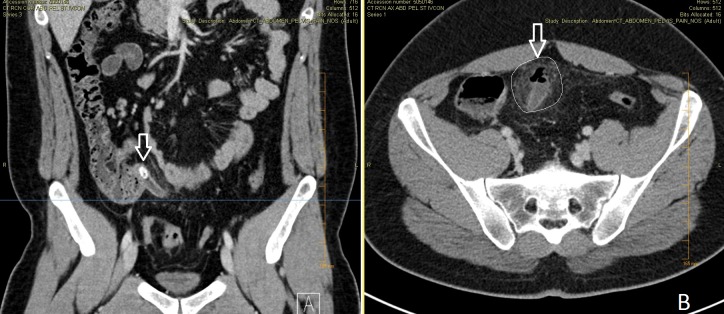
Computed tomography scan (CT) of abdomen and pelvis Coronal view (image A) demonstrating a 1.2 cm obstructing appendicolith within the proximal appendix (arrow). Axial view (image B) showing fluid-filled, dilated distal appendix with periappendiceal inflammation and scattered foci of free air concerning for perforated appendicitis (outlined area and arrow).

The initial physical examination revealed high body temperature of 40 °C, sinus tachycardia of 115 bpm, and systolic blood pressures ranging between 120 to 130 mmHg. He had diffuse abdominal tenderness. Laboratory studies showed white blood cell count (17.1 K/uL), hemoglobin (15.5 g/dL), platelet count (218 K/uL), blood urea nitrogen (9 mg/dL), and creatinine (1.3 mg/dL). Computed tomography (CT) scans of the abdomen and pelvis demonstrated perforated appendicitis with proximal obstructing fecalith (Figure 1).

The patient was resuscitated with crystalloids, started on broad spectrum intravenous antibiotics, and then taken to the operating room for an appendectomy. Intraoperative findings included fecal spillage from appendiceal tip, purulent ascites, and the indurated omentum adherent to appendix. Standard laparoscopic appendectomy was performed. The duration of the operation was one hour with an estimated blood loss of 5 mL. The patient received 3500 mL of crystalloid intraoperatively, in addition to 2000 mL of crystalloid administered in the ED. At the end of the operation, the patient was extubated, and transferred to the floor.

**Figure 2:**
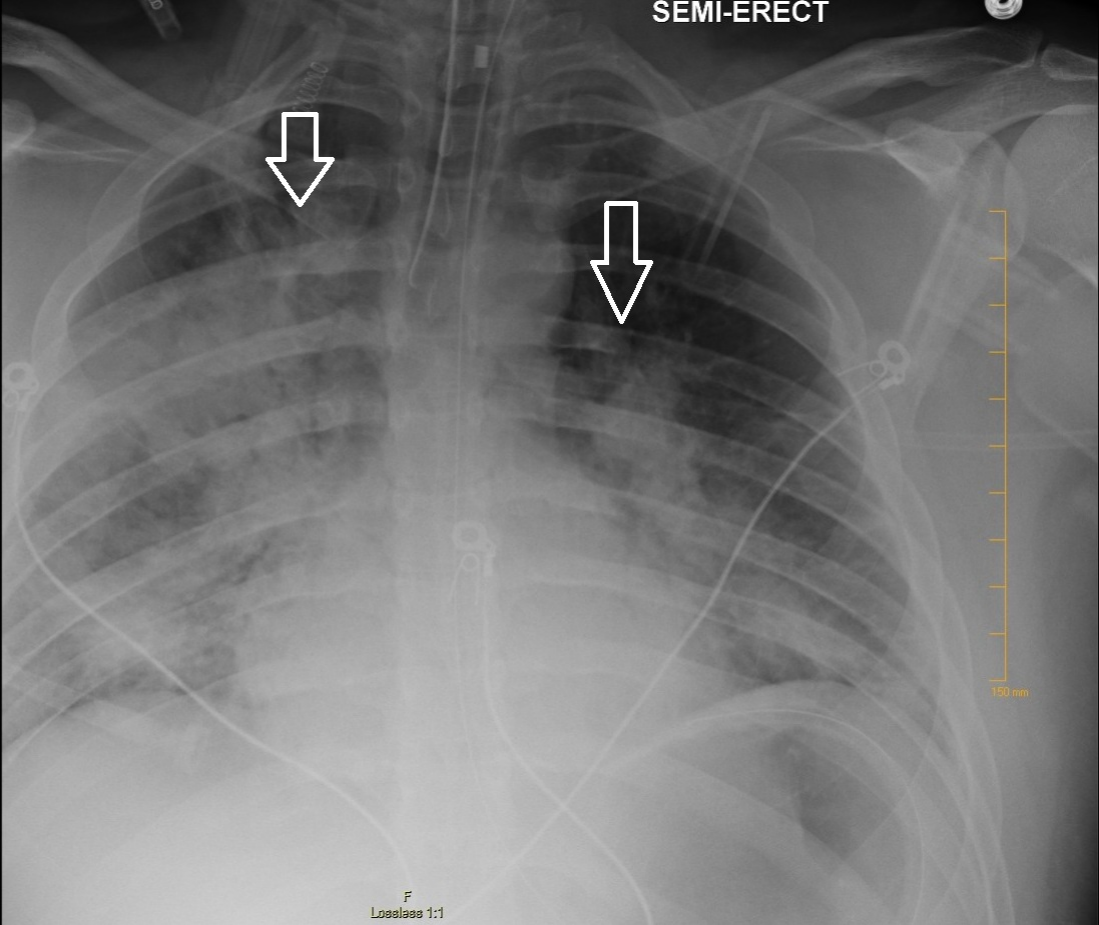
Chest radiography demonstrating bilateral hilar opacities (arrows)

On postoperative day two, patient developed acute hypoxic respiratory failure with severe adult respiratory distress syndrome (ARDS), with the arterial partial pressure of oxygen to the fraction of inspired oxygen (PaO2/FIO2) ratio less than 100 mmHg. Chest radiography obtained revealed bilateral hilar opacity (Figure 2). There was no evidence of heart failure. He was managed on the ventilator with low tidal volume and high positive end-expiratory pressure (PEEP) lung protective setting, following the acute respiratory distress syndrome network (ARDSNet) protocol. He was extubated on postoperative day six and discharged home on postoperative day eight.

Pathology of the appendix revealed a 6.2 cm x 1.1 cm appendix with attached mesoappendix. The cross-section of the distal tip was perforated and necrotic, with proximal obstruction by a fecalith.

## Discussion

ARDS is a diffuse, sudden-onset inflammatory process involving both lungs that results in increased pulmonary vascular permeability, decreased lung compliance, and alveolar edema and damage [1]. This disorder usually arises due to conditions that cause a widespread inflammatory process such as sepsis, aspiration, pneumonia, severe trauma, or massive transfusion [2]. Patients with ARDS present with a myriad of signs and symptoms, all related to the profound and sudden decrease in both lung compliance and effective gas exchange. Severe respiratory distress ensues, causing the patient to experience dyspnea, tachypnea, tachycardia, and progressive hypoxemia. These findings (along with possible cough, chest pain, and diffuse crackles) usually present within six to 72 hours after an inciting event, and generally tend to worsen rapidly [2].

According to the Berlin definition (revised in 2012), the diagnosis of ARDS requires four criteria to be met: hypoxemia refractory to oxygen therapy, diffuse bilateral pulmonary infiltrates consistent with pulmonary edema on chest radiograph (CXR) or computed tomography (CT) scan, respiratory failure not fully explained by cardiac failure, and respiratory symptoms that began within one week of a known clinical insult, or new or worsening symptoms during the past week. Once all four of these criteria are met, and cardiogenic pulmonary edema as well as other causes of acute hypoxemic respiratory failure are excluded, the ratio of arterial oxygen tension to fraction of inspired oxygen (PaO2/FiO2) can be used to determine the severity of the ARDS. A PaO2/FiO2 of 200 mmHg to less than or equal to 300 mmHg is mild, 100 mmHg to 200 mmHg is moderate, and less than 100 mmHg is severe [1].

The incidence of ARDS is approximately 190,000 cases a year in the United States; this has not substantially changed in the last few decades [3-4]. ARDS is more common in older people, with an incidence of 306 per 100,000 person-years among individuals ages 75 to 84 compared to 16 per 100,000 person-years among individuals ages 15 to 19 [3]. It is a commonly seen complication among patients in intensive care units, seen in approximately 10 to 15 percent of admitted patients, with up to 23 percent of intubated patients meeting the diagnostic criteria for ARDS [5-6]. The therapeutic options for ARDS include lung protective ventilation, neuromuscular blockade, prone positioning, inhaled vasodilators, corticosteroid, recruitment maneuvers, and extracorporeal life support [7]. The mortality of adult ARDS is still greater than 40 percent [4].

In our case, the patient developed severe septic ARDS secondary to perforated appendicitis, requiring mechanical ventilation with low tidal volume (six to eight mL/kg) and PEEP lung protective setting in accordance with the Acute Respiratory Distress Syndrome Network (ARDSNet) protocol [8]. His history of asthma, as well as the amount of crystalloid resuscitation administered, were contributory to the development of his pulmonary complication. He was administered about 5500 mL of crystalloid within the first six hours of presenting to the hospital, in the absence of septic shock. A recent, large multicenter cohort study of septic adult patients, by Seethala, et al. to identify risk factors readily detectable during the first six hours of hospital presentation associated with the development of ARDS and to examine the association of fluid administration during the first six hours and ARDS, showed that the risk factors within six hours of presentation associated with septic ARDS include: the acute physiology and chronic health evaluation (APACHE) II score, presence of shock, pulmonary source of infection, pancreatitis, and presence of an acute abdomen. It also showed that in septic patients without shock, the amount of fluid infused during the first six hours of hospital presentation was associated with developing ARDS [9].

## Conclusions

The development of severe ARDS after a uncomplicated laparoscopic appendectomy in a healthy adult is uncommon. In the case presented, the presence of sepsis, an acute abdomen, and the amount of fluid administered within the first six hours of presentation, in the absence of shock, contributed to the development of ARDS in the postoperative period. The treatment of septic ARDS include ensuring source control and providing supportive care.
